# deepMNN: Deep Learning-Based Single-Cell RNA Sequencing Data Batch Correction Using Mutual Nearest Neighbors

**DOI:** 10.3389/fgene.2021.708981

**Published:** 2021-08-10

**Authors:** Bin Zou, Tongda Zhang, Ruilong Zhou, Xiaosen Jiang, Huanming Yang, Xin Jin, Yong Bai

**Affiliations:** ^1^BGI-Shenzhen, Shenzhen, China; ^2^College of Life Science, University of Chinese Academy of Sciences, Beijing, China; ^3^James D. Watson Institute of Genome Sciences, Hangzhou, China; ^4^School of Medicine, South China University of Technology, Guangzhou, China; ^5^Guangdong Provincial Key Laboratory of Human Disease Genomics, Shenzhen Key Laboratory of Genomics, BGI-Shenzhen, Shenzhen, China

**Keywords:** scRNA-seq data integration, batch effect correction, residual network, mutual nearest neighbor, deep learning

## Abstract

It is well recognized that batch effect in single-cell RNA sequencing (scRNA-seq) data remains a big challenge when integrating different datasets. Here, we proposed deepMNN, a novel deep learning-based method to correct batch effect in scRNA-seq data. We first searched mutual nearest neighbor (MNN) pairs across different batches in a principal component analysis (PCA) subspace. Subsequently, a batch correction network was constructed by stacking two residual blocks and further applied for the removal of batch effects. The loss function of deepMNN was defined as the sum of a batch loss and a weighted regularization loss. The batch loss was used to compute the distance between cells in MNN pairs in the PCA subspace, while the regularization loss was to make the output of the network similar to the input. The experiment results showed that deepMNN can successfully remove batch effects across datasets with identical cell types, datasets with non-identical cell types, datasets with multiple batches, and large-scale datasets as well. We compared the performance of deepMNN with state-of-the-art batch correction methods, including the widely used methods of Harmony, Scanorama, and Seurat V4 as well as the recently developed deep learning-based methods of MMD-ResNet and scGen. The results demonstrated that deepMNN achieved a better or comparable performance in terms of both qualitative analysis using uniform manifold approximation and projection (UMAP) plots and quantitative metrics such as batch and cell entropies, ARI F1 score, and ASW F1 score under various scenarios. Additionally, deepMNN allowed for integrating scRNA-seq datasets with multiple batches in one step. Furthermore, deepMNN ran much faster than the other methods for large-scale datasets. These characteristics of deepMNN made it have the potential to be a new choice for large-scale single-cell gene expression data analysis.

## Introduction

High-throughput single-cell RNA sequencing (scRNA-seq) has enabled the gene expression profiling of a large number of individual cells at a single-cell resolution, offering unprecedented insights into the transcriptomic characterization of cell heterogeneity and dynamics (Stegle et al., 2015; Consortium, 2018; Han et al., 2018; Svensson et al., 2018). Considerable efforts have been made over the past decade to promote the rapid development of this technology, leading to massive single-cell gene expression data compiled from different experiments at different times and even with various sequencing platforms. However, like other sequencing technologies, these differences inevitably cause an unexpected batch effect due to the technical or biologically irrelevant variations across batches (Goh et al., 2017; Tran et al., 2020). The batch effect in the scRNA-seq data has been plaguing downstream analysis as it may interrupt the gene expression patterns. Consequently, the issue of batch effect may lead to a spurious conclusion when jointly investigating the comprehensive biological process of cells on the basis of integrating multiple datasets. Hence, batch effect correction is crucial for analyzing scRNA-seq data, allowing investigators to capture the intrinsically biological features across batches.

Currently, a myriad of batch effect correction algorithms has been proposed to tackle the problem (Tran et al., 2020). MNNCorrect (Haghverdi et al., 2018) assumed the orthogonality of batch effect to the biological manifold and corrected batch effect by calculating average difference in the high-dimensional gene expression space between similar cells across batch pairs (called mutual nearest neighbors, MNNs). Yet due to its high consumption of memory usage and CPU runtime, a number of methods were further developed to enhance the performance, for example, fastMNN (Haghverdi et al., 2018) and Seurat Integration (Seurat V3) (Stuart et al., 2019) followed the MNN scheme to carry out MNN search in a subspace by applying principal component analysis (PCA) and canonical correlation analysis (CCA), respectively. Scanorama (Hie et al., 2019) performed a faster approximate nearest neighbor search in the low-dimensional space computed by the randomized singular value decomposition. BBKNN (Polański et al., 2020) found MNNs in a low-dimensional, reduced space by computing k nearest neighbors and transformed the neighbor information into connectivity to construct a graph that linked all cells across batches. Harmony (Korsunsky et al., 2019) projected cells across different batches into a PCA space, followed by iteratively grouping similar cells into multiple clustering while simultaneously maximizing the diversity of batches within each cluster. LIGER (Welch et al., 2019) employed integrative non-negative matrix factorization to reduce the dimension and identified shared and batch-specific features across datasets. It then detected joint clusters and normalized the factor loading quantiles to perform batch correction. scMerge (Lin et al., 2019) constructed a graph connecting mutual nearest clusters between batches to remove batch effects.

Deep learning-based methods for single-cell analysis have experienced a tremendous progress in recent years and were already applied to remove batch effects in scRNA-seq data, for instance, MMD-ResNet (Shaham et al., 2017) has attempted to remove batch effect by minimizing the maximum mean discrepancy (MMD) using residual neural networks. BERMUDA (Wang et al., 2019) sought to remove batch effect locally based on MMD loss between similar cell clusters using an autoencoder structure. scGen (Lotfollahi et al., 2019) corrected batch effect based on the distributions of the cells that were inferenced from a reference dataset using a variational autoencoder model. However, scGen was a supervised method that required cell types in advance. scGAN (Bahrami et al., 2020) labeled multiple batches of the input cells that were represented in latent embedding space using a generative adversarial network model.

Although several batch correction methods are available, most of them struggle with excessive running time or resource requirements, which are likely to be further exacerbated as the cell numbers of scRNA-seq experiments continue growing. In this study, we propose deepMNN, a deep learning-based scRNA-seq batch correction model using MNN. We first identified MNN pairs among batches in a PCA subspace. A residual-based batch correction network was then constructed and employed to remove batch effects based on these MNN pairs. The overall loss of deepMNN was designed as the sum of a batch loss and a weighted regularization loss. The batch loss was used to compute the distance between cells in MNN pairs in the PCA subspace, while the regularization loss was to make the output of the network similar to the input. We compared the performance of deepMNN with state-of-the-art batch correction methods, including the widely used methods of Harmony, Scanorama, and Seurat V4, as well as the recently developed deep learning-based methods of MMD-ResNet and scGen. To comprehensively investigate the performance of these methods, we employed different scRNA-seq datasets under various scenarios, such as datasets with non-identical cell types, datasets with multiple batches, and large-scale datasets. In addition to qualitative analysis using uniform manifold approximation and projection (UMAP) plots, we calculated three metrics to quantitatively compare their performance on batch correction, including batch and cell type entropies, adjusted rand index (ARI) F1 score, and average silhouette width (ASW) F1 score. The experiment results showed that, in comparison to other correction methods, deepMNN not only reached a better or comparable performance in terms of the quantitative metrics and running time but also allowed for integrating scRNA-seq datasets with multiple batches in one step.

## Materials and Methods

### Architecture of deepMNN

**FIGURE 1:**
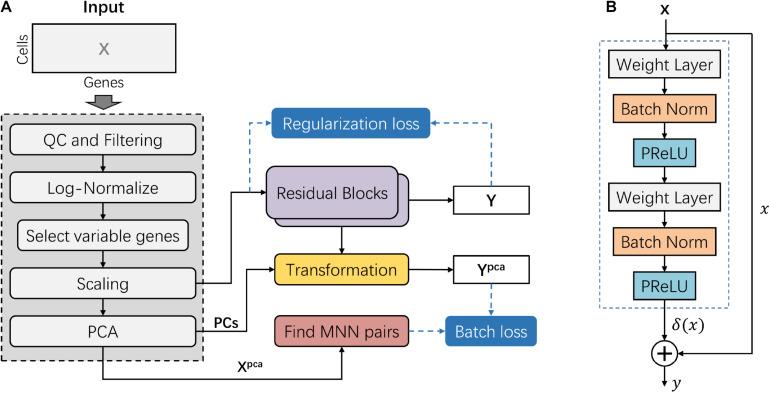
Overview of the deepMNN framework. **(A)** Illustration of the deepMNN workflow that was comprised of data pre-processing, principal component analysis transformation, mutual nearest neighbor pair search, batch correction network with a stack of two residual blocks, and calculation of batch loss and regularization loss. **(B)** Residual block comprised of two sequences of three consecutive layers, including the weight layer, batch normalization layer, and PReLU activation layer.

The deepMNN encompassed two main steps: pre-processing and batch correction (Figure 1A). The pre-processing step followed the standard workflow for scRNA-seq data analysis in Scanpy (Wolf et al., 2018), such as quality control (QC), filtering, normalization, identification of highly variable genes, scaling, and linear dimensional reduction using PCA. The dimensional-reduced data *X*^*pca*^ was used to find MNN pairs among the different batches. In the batch correction step, the scaled data was fed into the batch correction network, and the output was further transformed into the PCA subspace. Here, the batch correction network was formed by the stack of two residual blocks. Each residual block received an input *x* and computed output *y* = *x* + δ(*x*), where δ(*x*) was the output of the residual block (Figure 1B). The batch loss measured the distance between cells in MNN pairs in the PCA subspace. We also used a regularization loss to make the output of batch correction network resemble the input.

### Data Pre-processing

The steps of data pre-processing for scRNA-seq data included (1) QC and filtering, which was performed to remove the unwanted cells based on user-defined criteria, (2) normalization, the gene expression measurements for each cell were normalized by the total expression, followed by multiplication of a scale factor of 10,000, and (3) log-transformation, the normalized data was processed using log-transformation. Subsequently, 2,000 highly variable genes (HVGs, i.e., genes exhibiting high cell-to-cell variation in the dataset) were identified. We then scaled the data by calculating the z-score for each gene expression to have zero mean and unit variance. It should be noted that the z-score values exceeding the standard deviation of 10 were clipped. Next, we applied PCA on the scaled data and reduced the dimension using the first 50 principal components (PCs) empirically. The resulting matrix *X*^pca^ was further used to find MNN pairs across different batches. In addition, the first 50 PCs were also used to reduce the dimension of the outputs from the batch correction network as well (Figure 1A).

### Searching for MNN Pairs Among Batches

To find MNN pairs across batches, deepMNN searched 20 nearest neighbors for every cell in one batch from the remaining other batches in the dimensional-reduced PCA subspace. After repeating this process for all batches, we identified MNN pairs where a cell in one batch is the nearest neighbor of a cell in another batch and *vice versa*. Since the computational load of nearest neighbor queries was exponential in the size of the dataset, we improved the efficiency of our method using an approximate nearest neighbor searching algorithm that was implemented in the Annoy package^1^.

### Batch Correction Network

Inspired by the well-known residual network, the batch correction network was formed by the stack of two residual blocks. A residual block received an input *x* (or the output of the previous block) and computed output *y* = *x* + δ(*x*), where δ(*x*) is a residual term resulting from two sequences of three consecutive layers: weight layer, batch normalization layer, and PReLU activation layer (Figure 1B). The first weight layer in a residual block had 2 × *d* nodes, while the second weight layer had *d* nodes, where *d* is the input dimension of the residual block.

In our work, the initial input into the batch correction network was the scaled data with 2,000 selected HVGs. Consequently, the number of nodes in the first and the second weight layers of the first residual block was 4,000 and 2,000, respectively. The number of nodes in the two weight layers of the second residual block was correspondingly the same as that in the first residual block. Therefore, the number of nodes in the output layer of the batch correct network was 2,000.

### Loss Function

There were two types of losses in this study: (1) the batch loss that was the sum of the Euclidean distances between cells in the MNN pairs and (2) the regularization loss aimed to make the output of the network similar to the input.

To compute the batch loss, we first calculated the dimensional-reduced vector $Y_{i}^{\text{pca}}$ for cell *i* as follows:

$$Y_{i}^{\text{pca}}=Y_{i}\cdot \text{PCs}$$

where *Y_i_* is the output of the batch correction network for cell *i*; PCs are the first 50 principal components as described in section “Data Pre-processing.” Suppose two cells *i* and *j* were in the MNN pair *k* and, thus, denoted as $Y_{i_{k}}^{\text{pca}}$ and $Y_{j_{k}}^{\text{pca}}$, respectively. Then, the batch loss *L*_*b*_ can be written as follows:

$$L_{\text{b}}=\sum_{k}{||Y_{i_{k}}^{\text{pca}}-Y_{j_{k}}^{\text{pca}}||}_{2}$$

where ${||Y_{i_{k}}^{\text{pca}}-Y_{j_{k}}^{\text{pca}}||}_{2}$ represents the Euclidian distance between cells *i* and *j* in the MNN pair *k*, *k* = 1,2,3,…, *K*, and *K* is the total number of MNN pairs.

We hypothesized that the cells in an MNN pair had the same cell type, their distance should be small when no batch effect existed, and hence the batch loss was used to remove the batch effect between different batches. However, if the batch correction network had a zero vector output, the batch loss should have been zero, which was not our expectation. As such, we further utilized a regularization loss to make the output of the network not far away from the input.

The regularization loss *L*_r_ was defined as the sum of the Euclidian distances between the output and the input of the batch correction network.

$$L_{\text{r}}=\sum_{i}{||Y_{i}-X_{i}||}_{2}$$

where *Y_i_* is the output of the batch correction network of cell *i*, and *X_i_* is the cell *i* in the scaled data with 2,000 HVGs.

Finally, the overall loss of deepMNN was defined as the combination of a batch loss and a weighted regularization loss:

$$\text{L}=L_{\text{b}}+\alpha \cdot L_{\text{r}}$$

The value of α was set as 0.001 in our experiments.

### Hyperparameters for Training deepMNN

We trained the deepMNN batch correction network *de novo* with default initialization of weights as provided by the PyTorch library (version 1.6.0). We employed the Adam optimizer (Kingma and Ba, 2014) with default parameters β_1_ = 0.9 and β_2_ = 0.999 and a batch size of 1,024 for all experiments. The maximum number of epochs was set as 200. The training procedure would stop early when the total loss did not decrease for 10 consecutive epochs. The learning rate (LR) was initialized as 0.1 and decayed by 0.8 every 20 epochs. In general, the hyperparameters of the network were manually optimized. We searched primarily over the residual block structure, empirically chose the number of the residual blocks, and manually tuned the LR to obtain optimal performance.

### Batch Correction Through Other Methods

Three widely used methods of Harmony, Scanorama, and Seurat V4 and two deep learning-based methods of MMD-ResNet and scGen were used to compare the performance on batch correction with deepMNN.

We first applied the same data pre-processing as described in section ‘‘Data Pre-processing’’ for all these methods, including QC and filtering, normalization, and log-transformation. For Harmony, the first 50 PCs were determined by applying PCA on the pre-processed data, followed by utilization of the RunHarmony function in its R package (version 0.1.0) to conduct the batch correction experiments. The parameters of maximum clusters and maximum iterations were set as 50 and 100, respectively. For Scanorama, we first identified 2,000 HVGs after data pre-processing and then employed its Python implementation (version 1.7.1) to perform the experiments with default parameter settings. For Seurat V4, we followed the Seurat integration workflow recommended by the Seurat package (version 4.0.3). Briefly, we first selected 2,000 HVGs from the pre-processed data and then computed the anchors using the FindIntegrationAnchors function, followed by integration of the batches using the IntegrateData function to accomplish the experiments. For MMD-ResNet, the PyTorch implementation^2^ was used to perform the experiments. After data pre-processing and dimension reduction using PCA, we selected the first 50 PCs to train the MMD-ResNet model with default hyperparameters but with a batch size of 256. The training stopped when the loss did not decrease for five consecutive epochs. For scGen, we used the PyTorch implementation (version 2.0.0) to carry out the experiments in our work. We selected the top 7,000 HVGs by default from the pre-processed data to train the scGen model with default hyperparameters except for epochs of 100 and a batch size of 32.

To assess the performance of each method including deepMNN, the top 50 PC vectors extracted from the batch-corrected expression matrix were used for the calculation of evaluation metrics and visualization.

### Datasets

#### Human Peripheral Blood Mononuclear Cell

The data included two batches of human peripheral blood mononuclear cells (PBMCs) from two healthy donors, which were generated by the 3′ and 5′ Genomics protocols, respectively (Zheng et al., 2017). The data and the cell type annotated by Polański et al. (2020) were downloaded from ftp://ngs.sanger.ac.uk/production/teichmann/BBKNN/PBMC.merged.h5ad. We excluded cells without annotation and only retained common genes, resulting in nine different cell types for a total of 8,098 cells in the 3′ batch and 7,378 cells in the 5′ batch, each with 17,430 genes.

#### Human Pancreas

The data consisted of five published pancreas datasets: Baron (GSE84133) (Baron et al., 2016), Muraro (GSE85241) (Muraro et al., 2016), Segerstolpe (E-MTAB-5061) (Segerstolpe et al., 2016), Wang (GSE83139) (Wang et al., 2016), and Xin (GSE81608) (Xin et al., 2016), generated using inDrop, CEL-Seq2, SMART-Seq2, SMARTer, and SMARTer protocols, respectively. The data batches and annotations were downloaded from https://hemberg-lab.github.io/scRNA.seq.datasets/human/pancreas/. We removed the cells annotated with unknown cell types and only retained the genes detected in all batches. As a result, there were 15 different cell types for a total of 14,767 cells, each with 15,558 genes.

#### Human Cell Atlas

The Human Cell Atlas (HCA) dataset was downloaded from https://github.com/immunogenomics/harmony2019/tree/master/data/figure3, processed by Korsunsky et al. (2019). This data had two batches, including 275,264 bone marrow cells and 253,024 cord blood cells, respectively (Li et al., 2018). 10× Genomics protocol was used to generate the data, and 24,823 genes were acquired for each cell. We removed the cell types whose number of cells was less than 200, resulting in 22 different cell types for a total of 528,014 cells.

### Evaluation Metrics for Batch Correction

To assess the batch correction performance of deepMNN and other methods as described above, we calculated three types of metrics, batch and cell type entropies (Chazarra-Gil et al., 2021), ARI F1 score (Hubert and Arabie, 1985; Tran et al., 2020), and ASW F1 score (Rousseeuw, 1987; Tran et al., 2020).

#### Batch and Cell Type Entropies

The entropies of batch and cell type can be used to measure batch mixing and cell type separation. To compute the batch and cell type entropies, we first constructed a KNN graph where each cell was a node and connected to its 20 nearest neighbors. Then, the batch entropy $E_{i}^{\text{b}}$ and cell type entropy $E_{i}^{\text{c}}$ for cell *i* were calculated as follows:

$$P_{i\text{b}}=\frac{{\text{N}}_{i\text{b}}}{{\text{N}}_{i}}$$

$$E_{i}^{\text{b}}=-\frac{1}{B}\sum_{\text{b}}P_{i\text{b}}\text{log}\left(P_{i\text{b}}\right)$$

$$P_{i\text{c}}=\frac{{\text{N}}_{i\text{c}}}{{\text{N}}_{i}}$$

$$E_{i}^{\text{c}}=-\frac{1}{C}\sum_{\text{c}}P_{i\text{c}}\text{log}\left(P_{i\text{c}}\right)$$

where N_*i*_ is the number of neighbors of cell *i* (N_*i*_ = 20 for each cell *i*), N_*i*b_ is the number of neighbors of cell *i* with batch b, N_*i*c_ is the number of neighbors of cell *i* with cell type c, and *B* and *C* are the number of batches and the number of cell types, respectively. A high batch entropy indicates a homogeneous mixture of different batches, while a low cell type entropy suggests that the cell types remain separate.

#### Adjusted Rand Index F1 Score

The rand index (RI) measures the similarity of results between two clustering methods. It is useful to compare the true label distribution with the clustering prediction and, therefore, can also be applied to measure batch mixing and cell type separation. The RI is defined as:

RI=a+b(n2)

where *a* is the number of pairs of cells with the same true label that belongs to the same cluster, *b* is the number of pairs of cells with a different true label that are assigned to different clusters, and (n2) is the number of unordered pairs in a set of *n* cells. To ensure a value close to 0 for random labeling, the RI score is “adjusted for chance,” which gives the ARI:

$$ARI=\frac{RI-E\left(RI\right)}{max\left(RI\right)-E\left(RI\right)}$$

where *E*(*RI*) and *max*(*RI*) are the expectation and maximum of RI, respectively. The ARI score ranges from −1 to 1. A positive high ARI score suggests that the result of clustering prediction is much consistent with the true label distribution.

To obtain the ARI score, we first applied the k-means algorithm to generate cluster labels for comparison against batch labels and cell type labels. We then randomly selected 80% of cells and calculated the ARI scores for batch and cell type. This procedure was repeated 20 times to ensure stability. The batch ARI score and cell type ARI score were further normalized into an interval of [0, 1], which were denoted as ARI_batch_norm_ and ARI_celltype_norm_, respectively. Finally, the ARI F1 score was defined as:

$$F1_{ARI}=\frac{2\left(1-ARI_{batch\_norm}\right)\left(ARI_{celltype\_norm}\right)}{1-ARI_{batch\_norm}+ARI_{celltype\_norm}}$$

The ARI F1 score is the harmonic mean of the ARI batch score and the ARI cell type score. As a combined measurement of batch mixing and cell type separation, a higher ARI F1 score indicates a better performance of the batch correction method.

#### Average Silhouette Width F1 Score

The silhouette score measures how well a cell lies within its own cluster in comparison with other clusters. It is defined as:

$$s_{i}=\frac{\left(b_{i}-a_{i}\right)}{max\left(a_{i},b_{i}\right)}$$

where *a_i_* is the average distance between cell *i* and other cells in its cluster, and *b_i_* is the average distance between cell *i* and the cells in its nearest cluster. The silhouette score is between −1 and 1. A positive high silhouette score suggests that the cell is close to its own cluster but discrepant to other clusters. The ASW score over the entire dataset is then given by:

$$\text{ASW}=\frac{1}{n}\sum_{i}s_{i}$$

where *n* is the total number of cells in the dataset. The ASW score indicates whether the clusters are well separated and, hence, can also be used to evaluate the performance of the batch correction methods.

Like the calculation of the ARI score, we randomly selected 80% of cells to compute the ASW batch score and the ASW cell type score and repeated this procedure 20 times. We normalized the ASW batch score and the ASW cell type score into an interval [0, 1]. The ASW F1 score was then obtained by calculating the harmonic mean of the normalized ASW batch score and the normalized ASW cell type score as follows:

$$F1_{ASW}=\frac{2\left(1-ASW_{batch\_norm}\right)\left(ASW_{celltype\_norm}\right)}{1-ASW_{batch\_norm}+ASW_{celltype\_norm}}$$

The ASW F1 score is a combined metric to assess batch mixing and cell type separation. A higher ASW F1 score indicates better performance.

### Statistical Test and Visualization

The Mann–Whitney *U*-test with the Benjamini–Hochberg correction was applied to the ARI F1 scores and the ASW F1 scores to compare the performance on batch correction between deepMNN and other methods.

We used UMAP (Becht et al., 2019) implemented in the Scanpy library (version 1.6.0) to visualize our batch correction results with default parameters.

## Results

We utilized the three datasets of PBMCs with two batches, pancreas cells with five batches, and HCA cells with two batches (Table 1) to evaluate all batch correction methods under four different scenarios: identical cell types, non-identical cell types, multiple batches, and large datasets.

**TABLE 1:** Single-cell RNA sequencing datasets used for evaluating deepMNN.

Dataset	Batch	Protocol	Number of cells
PBMC	10× 3′	10× Chromium Single Cell 3′ v2 chemistry	8,098
	10× 5′	10× Chromium Single Cell 5′ paired-end chemistry	7,378
Pancreas	Baron	inDrops	8,569
	Muraro	CelSeq2	2,122
	Segerstolpe	SMART-seq2	2,127
	Wang	SMARTer	457
	Xin	SMARTer	1,492
HCA	Bone Marrow	10×	275,264
	Cord blood	10×	253,024

The experiments were carried out on a workstation with four NVIDIA GeForce GTX 1080 Ti graphics cards, two Intel Xeon E5-2620 v4 CPUs, and 64G random access memory (RAM). We performed experiments for all methods in the CPU environment except the deep learning-based methods of deepMNN, scGen, and MMD-ResNet, for which a single GPU card was used.

### Scenario 1: Identical Cell Types

**FIGURE 2:**
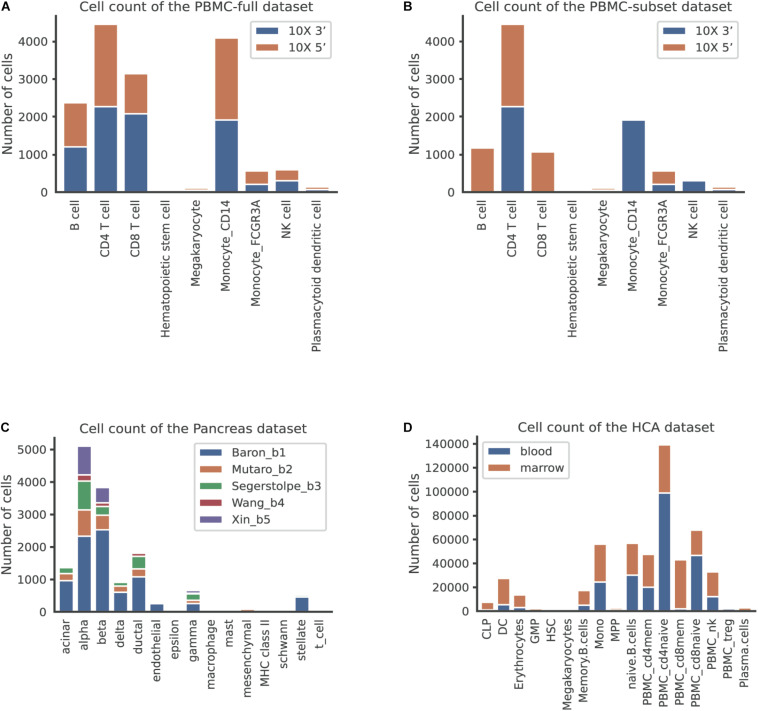
The number of cells per cell type across batches in different datasets. **(A)** The human peripheral blood mononuclear cell (PBMC) full dataset, corresponding to the scenario of identical cell types. **(B)** The PBMC subset dataset, corresponding to the scenario of non-identical cell types. **(C)** The pancreas dataset, corresponding to the scenario of multiple batches. **(D)** The human cell atlas dataset, corresponding to the scenario of large-scale datasets.

**FIGURE 3:**
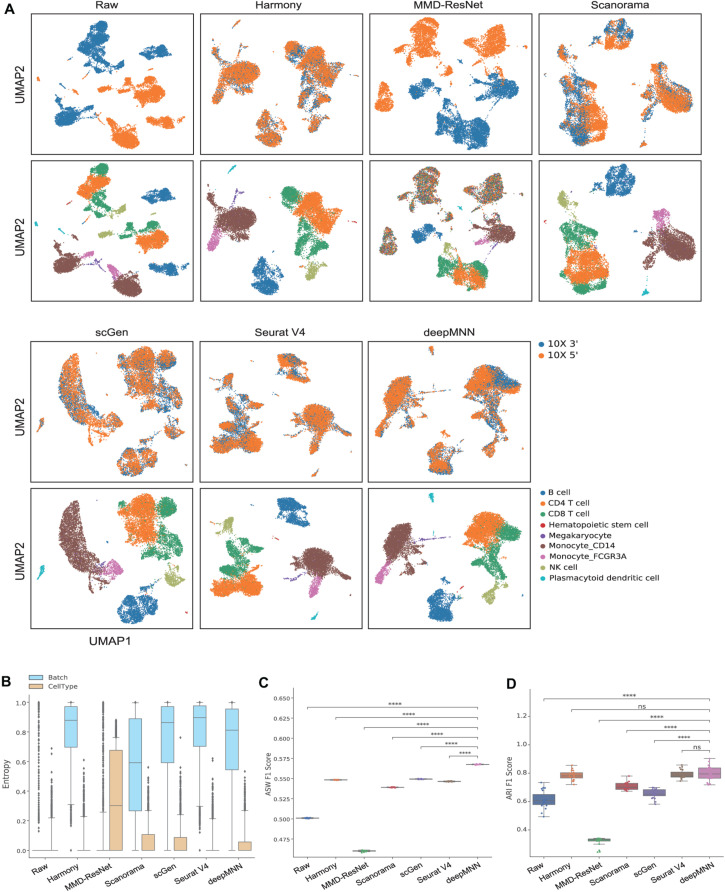
Comparison of batch effect correction methods for the human peripheral blood mononuclear cell dataset of identical cell types with two batches. **(A)** Qualitative evaluation of the raw data, Harmony, MMD-ResNet, Scanorama, scGen, Seurat V4, and deepMNN using UMAP. The cells were colored by batches on the top row and colored by cell type on the bottom row. **(B)** The batch and cell type entropies resulting from the batch correction methods. The plots show the median (line within box), 25th and 75th percentiles (box), 5th and 95th percentiles (whiskers), and outliers (diamond points). **(C)** The ASW F1 score resulting from different batch correction methods. **(D)** The ARI F1 scores resulting from different batch correction methods. ^****^p ≤ 0.0001.

We first used the PBMC dataset to evaluate the batch correction methods. This dataset was comprised of nine identical cell types and possessed a similar proportion of cells for each cell type between the two batches (Figure 2A). The UMAP plots depicted that all methods except MMD-ResNet successfully merged the common cells (Figure 3A). The deepMNN, Harmony, and Seurat V4 produced a distinct megakaryocyte cluster from other cell type clusters. By comparison, most megakaryocyte cells were mixed up with monocyte CD14 cells by Scanorama and scGen. Moreover, the CD8 cells located much closer within the compact clusters that resulted from deepMNN and Seurat V4. However, these cells scattered around the CD4 T cells in the clusters generated by Harmony, Scanorama, and scGen.

With regards to the batch and cell type entropies (Figure 3B), deepMNN achieved a comparable or a slightly lower batch entropy than Harmony, scGen, and Seurat v4, but higher than MMD-ResNet and Scanorama. A lower cell type entropy was reached by deepMNN compared to other methods except for Harmony and Seurat V4. As for the ASW F1 score (Figure 3C), deepMNN was significantly higher than the other methods (*p* < 0.00001). Furthermore, the results of the ARI F1 scores (Figure 3D) showed that the performance of deepMNN was comparable with that of Harmony and Seurat V4 and significantly better than all the other methods (*p* < 0.00001).

### Scenario 2: Non-identical Cell Types

**FIGURE 4:**
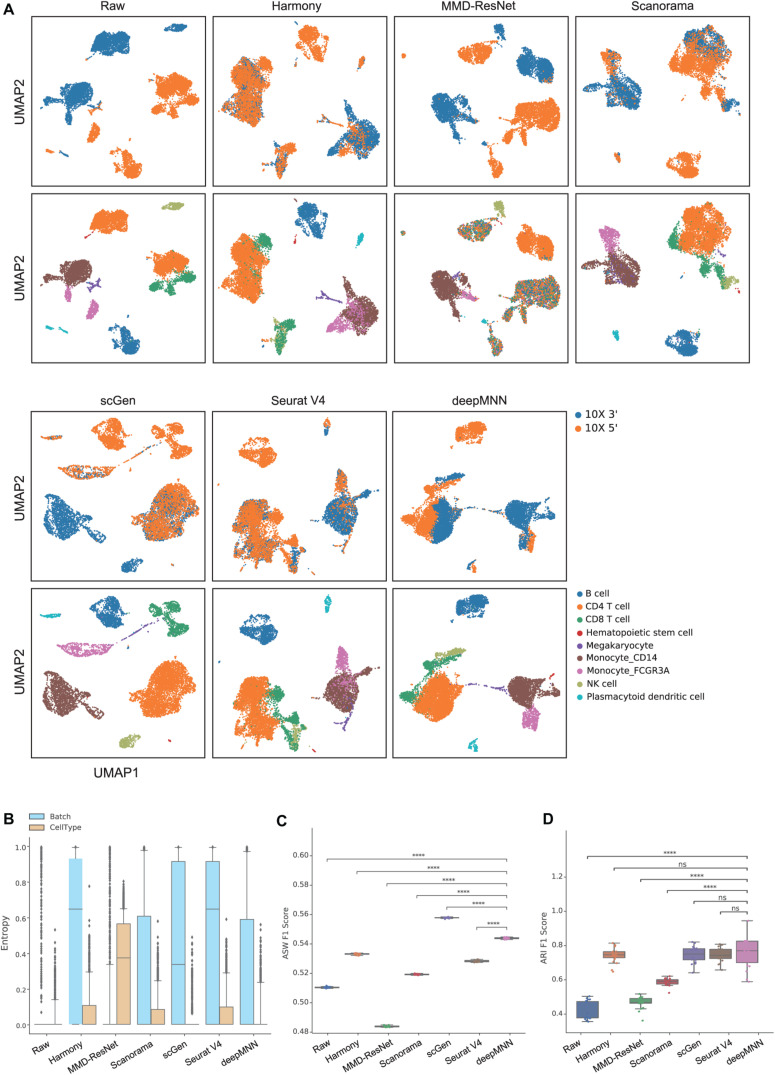
Comparison of batch effect correction methods for the human peripheral blood mononuclear cell dataset of non-identical cell types with two batches. **(A)** Qualitative evaluation of the raw data, Harmony, MMD-ResNet, Scanorama, scGen, Seurat V4, and deepMNN using UMAP. The cells were colored by batches on the top row and colored by cell type on the bottom row. **(B)** The batch and cell type entropies resulting from the batch correction methods. The plots show the median (line within box), 25th and 75th percentiles (box), 5th and 95th percentiles (whiskers), and outliers (diamond points). **(C)** The ASW F1 score resulting from different batch correction methods. **(D)** The ARI F1 scores resulting from different batch correction methods. ^****^p ≤ 0.0001.

To evaluate deepMNN under the scenario where batches had non-identical cell types, we downsampled the PBMC dataset using the following criteria: (1) the CD8 and B cells were removed from the 10× 3′ batch and (2) the monocyte CD14 and NK cells were removed from the 10× 5′ batch. As a result, the two batches had different cell types except for CD4, megakaryocyte, and monocyte FCGR3A cells (Figure 2B). Similar to the results from scenario 1, we observed that all the methods, except MMD-ResNet, merged the two batches (Figure 4A). The deepMNN, Harmony, scGen, and Seurat V4 produced well-separated clusters for megakaryocyte cells that, however, were mixed up with monocyte CD14 cells using Scanorama. Moreover, it was observed that the methods of Harmony, Scanorama, and Seurat V4 mixed up some CD8 T cells with CD4 T cells, some other CD8 T cells with NK cells, and some monocyte FCGR3A cells with monocyte CD14 cells. In contrast, all cell types were clearly distinguished by deepMNN except that only a few of CD8 T cells were mixed up with NK cells.

Regarding the batch and cell type entropies, deepMNN was one of the methods that obtained the lowest cell entropy (Figure 4B). It had a lower batch entropy than Harmony, scGen, and Seurat v4 did. The ASW F1 score of deepMNN was lower than scGen but significantly higher than all other methods (*p* < 0.00001) (Figure 4C). No significant difference in the ARI F1 scores was observed between deepMNN and the methods of Harmony, scGen, and Seurat V4. However, deepMNN reached a significantly higher ARI F1 score than MMD-ResNet and Scanorama (*p* < 0.00001) (Figure 4D).

### Scenario 3: Multiple Batches

**FIGURE 5:**
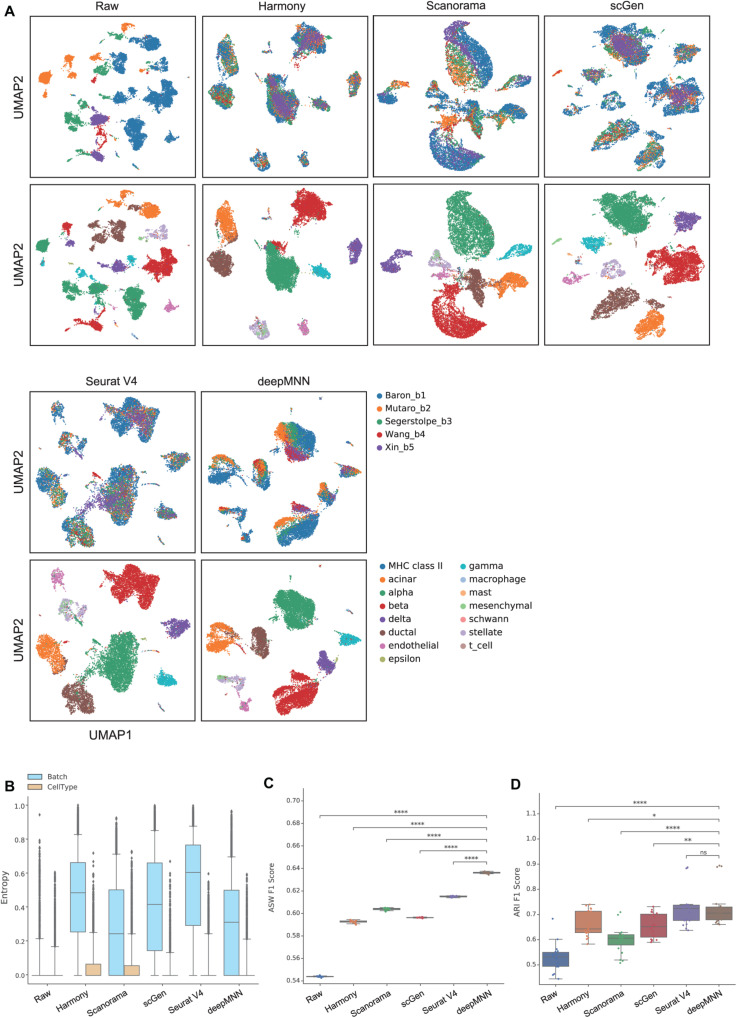
Comparison of batch effect correction methods for the pancreas datasets with five batches. **(A)** Qualitative evaluation of the raw data, Harmony, Scanorama, scGen, Seurat V4, and deepMNN using UMAP. The cells were colored by batches on the top row and colored by cell type on the bottom row. **(B)** The batch and cell type entropies resulting from the batch correction methods. The plots show the median (line within box), 25th and 75th percentiles (box), 5th and 95th percentiles (whiskers), and outliers (diamond points). **(C)** The ASW F1 score resulting from different batch correction methods. **(D)** The ARI F1 scores resulting from different batch correction methods. ^∗^0.01 < *p* ≤ 0.05, ^∗∗^0.001 < *p* ≤ 0.01, ^****^p ≤ 0.0001.

To assess the performance of deepMNN on a dataset with multiple batches, we employed the dataset of human pancreatic cells that consisted of five batches. The dataset had different numbers of cells and non-identical cell types between batches (Figure 2C). The UMAP plots demonstrated that Harmony, scGen, and Seurat v4 can merge all batches, while deepMNN and Scanorama were more likely to make cell-specific clusters close together (Figure 5A). Interestingly, all methods appeared to have maintained a relatively good cell type separation.

For the evaluation metrics, deepMNN obtained a lower batch entropy than Harmony, scGen, and Seurat V4 and was one of the methods that achieved the lowest cell entropy (Figure 5B). It reached a significantly higher ASW F1 score compared to the other methods (*p* < 0.00001) (Figure 5C). The ARI F1 score from deepMNN was also significantly higher than that from Harmony (*p* < 0.05), Scanorama (*p* < 0.00001), and scGen (*p* < 0.001) except for Seurat V4 (*p* > 0.05) (Figure 5D). Due to the bad performance of MMD-ResNet in the experiments using two-batch datasets as shown above, we did not evaluate the method of MMD-ResNet under this multiple-batch scenario.

### Scenario 4: Large-Scale Dataset

**FIGURE 6:**
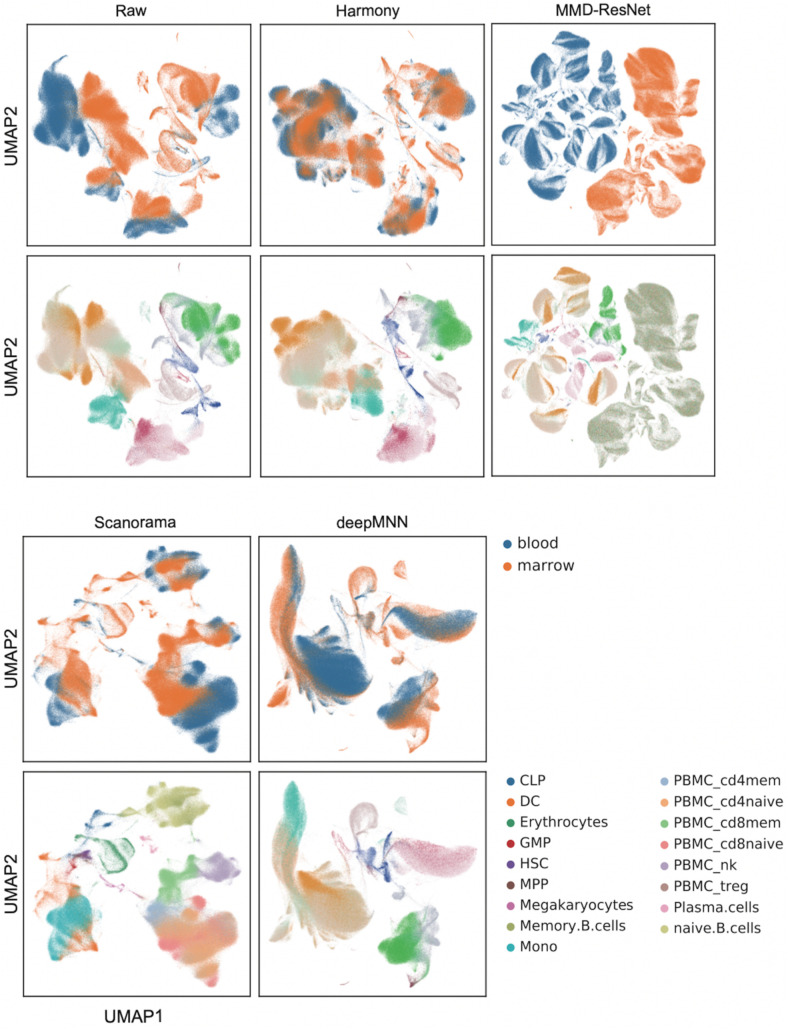
Evaluation of raw data, Harmony, MMD-ResNet, Scanorama, scGen, and deepMNN on the large-scale HCA dataset with 528,014 cells. The cells were colored by batches on the top row and colored by cell type on the bottom row.

We further evaluated the batch correction methods using the large-scale HCA dataset that was comprised of two batches, where one batch had 275,184 bone marrow cells, while another had 252,830 cord blood cells (Li et al., 2018; Figure 2D). Seurat V4 and scGen were not capable of running successfully on our server with 64GB RAM due to the exceedingly huge size of the dataset. The deepMNN took approximately 17 min to complete the process of batch effect correction, which was significantly faster than Harmony (∼35 min) and Scanorama (∼77 min). Since the computation of batch and cell type entropies required more than 1 TB RAM and the calculation of the ASW F1 score was unable to be completed within 48 h on our server, we did not provide the results of the quantitative metrics. However, it was observed that deepMNN, Harmony, and Scanorama were able to bring cell-specific clusters close together (Figure 6).

## Discussion

Batch effect poses a big challenge in scRNA-seq data analysis. In this study, we proposed deepMNN, a novel deep learning-based scRNA-seq batch correction method. The deepMNN was constructed by a residual-based batch correction network in conjunction with MNN pairs to remove batch effects in scRNA-seq data. The experiment results showed that deepMNN can successfully align different datasets under four scenarios such as identical cell types, non-identical cell types, multiple batches, and large-scale datasets. We compared the performance of deepMNN with state-of-the-art batch correction methods, including Harmony, Scanorama, and Seurat V4 as well as MMD-ResNet and scGen. The results demonstrated that deepMNN achieved a better or comparable performance in terms of both qualitative analysis using UMAP plots and quantitative metrics such as batch and cell entropies, ARI F1 score, and ASW F1 score as well as running time. Two review papers (Tran et al., 2020; Chazarra-Gil et al., 2021) reported that Harmony and Seurat were the best batch correction methods in most scenarios, which, in turn, suggested the high efficiency of deepMNN to correct batch effect.

The cell types and their proportions may be considerably different across batches. For MNN-based batch correction methods, such as MNNCorrect, Scanorama, and deepMNN, the MNN pairs across batches need to be computed first. When two cells from two datasets were identified in an MNN pair, they were likely the same cell type. To remove the batch effect, traditional methods usually calculated reference vectors based on the identified MNN pairs and mapped one dataset to the space obtained from the reference dataset. By comparison, deepMNN applied a batch correction network that was formed by the stack of two residual blocks for batch removal. Since the residual block contained a residual term δ(*x*) and an identity term *x*, deepMNN can easily learn a representation similar to the identity term. In addition, the distributions of the same cell types from different batches were theoretically close to each other, and the discrepancy may be introduced by the batch effect. Thus, the residual structure of deepMNN attempted to learn a representation for the identity term, and the residual term can be regarded as the batch effect.

Methods like Scanorama and Seurat V4 merged only two datasets at once and iterated the same procedure to accomplish the integration of multiple datasets. To our best knowledge, deepMNN was the first method to integrate multiple batches of scRNA-seq data in one step. After identifying MNN pairs among batches, we minimized the batch loss that measured the distance between cells in the MNN pairs, which can promote the network removing the multiple-batch effect simultaneously. It should be noted that the batch loss was not directly based on the output of the batch correction network. We applied the PCA instead to reduce the dimension of the output first and then calculated the distance between cells in the MNN pairs.

Compared to the state-of-the-art batch correction methods, deepMNN achieved almost significantly high ARI F1 scores and ASW F1 scores under the scenarios of identical cell types, non-identical cell types, and multiple batches. The scGen reached a higher ASW F1 score than deepMNN under the scenario of non-identical cell types. This was partially due to the feature of scGen that was a supervised learning method and required cell type labels. As for computation time, deepMNN was comparable with other methods when the dataset was small. However, it was significantly fast when dealing with large-scale datasets – for example, deepMNN spent around 17 min on batch correction for the 528k HCA dataset, while Harmony and Scanorama needed about 35 and 77 min, respectively. Korsunsky et al. (2019) compared the runtimes for different batch correction methods and reported Harmony as one of the fastest batch correction methods, which took 68 min on 500,000 cells. One reason for the ability of quick batch correction by deepMNN was likely that it removed batch effect in one step. Another reason might probably be that deepMNN converged fast and can complete batch correction within tens of epochs. In our experiments, deepMNN only required 50 to 100 epochs to accomplish the removal of batch effect. The last reason was partially due to the deep learning-based method of deepMNN that used GPU to speed up the computation. Seurat V4 and scGen cannot run on our 64GB server for the 528k HCA dataset due to their high RAM requirement.

**FIGURE 7:**
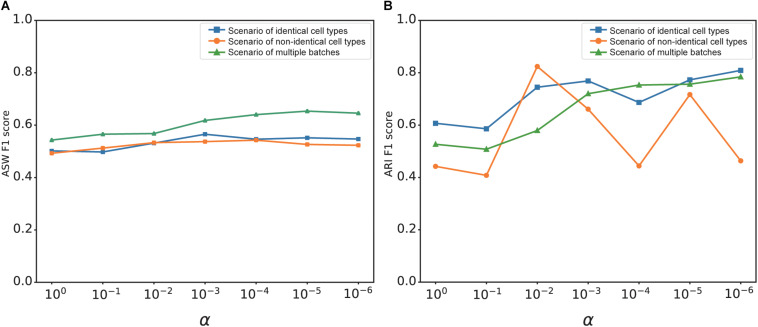
The effect of value changes in α on the batch correction performance of deepMNN under three scenarios of identical cell types, non-identical cell types, and multiple batches. **(A)** The ASW F1 scores *versus* various α values under different scenarios. **(B)** The ARI F1 scores *versus* various α values under different scenarios.

The overall loss of deepMNN was the sum of a batch loss and a weighted regularization loss that was controlled by the tradeoff parameter α. The use of regularization loss was to make the output of the network similar to the input and to prevent the output from being zero when no batches existed in a dataset. We investigated the effect of α on the batch correction performance of deepMNN in terms of the ARI F1 score and ASW F1 score under three different scenarios. Generally, the ASW F1 score tended to rise first and then declined with the decrease of α, and it reached almost the highest value when α was 0.001 under each of the three scenarios (Figure 7A). Although the ARI F1 score exhibited much fluctuation with the change of α, it can also have the highest value with α of 0.001 under the scenario of identical cell types (Figure 7B). Therefore, we chose 0.001 as the optimal value of parameter α.

One key limitation of our method was that deepMNN depended heavily on the identified MNN pairs. Only a small number of MNN pairs can be found when a handful of cells represented a shared biological state across batches, which was not sufficient to remove batch effects in the entire datasets effectively. On the other hand, even though a large number of MNN pairs have been identified but a low percentage of them have had the same cell types, deepMNN would result in a poor performance on batch correction. In our experiments, about 80–90% of MNN pairs had the same cell types. In the future, more reliable schemes of searching MNN pairs will be investigated. Another aspect of limitation in this study was related to the dimension reduction method. In this study, deepMNN used the PCA to project raw single-cell gene expression data into low-dimensional space. However, a previous study (Butler et al., 2018) demonstrated that PCA could intrinsically identify biologically irrelevant variations caused by technical effects. Other data embedding methods like CCA (Butler et al., 2018) and autoencoder (Li et al., 2020) would be further considered to improve the batch correction performance of deepMNN.

## Code Availability

The source code of deepMNN, including the experimental results of the study, can be found at https://github.com/zoubin-ai/deepMNN.

## Data Availability Statement

Publicly available datasets were analyzed in this study. These datasets can be found here: Human peripheral blood mononuclear cell (PBMC): ftp://ngs.sanger.ac.uk/production/teichmann/BBKNN/PBMC.merged.h5ad; Human pancreas: https://hemberg-lab.github.io/scRNA.seq.datasets/human/pancreas/; Human cell atlas (HCA): https://github.com/immunogenomics/harmony2019/tree/master/data/figure3.

## Author Contributions

BZ and YB conceived the algorithm and wrote the manuscript. BZ and RZ developed and performed the computational experiments. TZ performed the scRNA-seq experiments. BZ and XJa plotted the figures. YB, XJn, and HY supervised the study. All authors read and approved the final manuscript.

## Conflict of Interest

The authors declare that the research was conducted in the absence of any commercial or financial relationships that could be construed as a potential conflict of interest.

## Publisher’s Note

All claims expressed in this article are solely those of the authors and do not necessarily represent those of their affiliated organizations, or those of the publisher, the editors and the reviewers. Any product that may be evaluated in this article, or claim that may be made by its manufacturer, is not guaranteed or endorsed by the publisher.

## Footnotes

1
https://github.com/spotify/annoy

2
https://github.com/ushaham/batchEffectRemoval2020

## Abbreviations

MNN: mutual nearest neighbor
ARI: adjusted rand index
ASW: average silhouette width
PCA: principal component analysis
UMAP: uniform manifold approximation and projection
RAM: random access memory
GPU: graphics processing unit.
